# High Antioxidant Ability Confer Resistance to Atrazine in *Commelina communis* L.

**DOI:** 10.3390/plants10122685

**Published:** 2021-12-07

**Authors:** Juan Yang, Haiyan Yu, Hailan Cui, Jingchao Chen, Xiangju Li

**Affiliations:** 1 Key Laboratory of Weed Biology and Management, Institute of Plant Protection, Chinese Academy of Agricultural Sciences, Beijing 100193, China; yangjuan018@126.com (J.Y.); yuhaiyan2103@163.com (H.Y.); hlcui@ippcaas.cn (H.C.); jcchen@ippcaas.cn (J.C.); 2 Hebei Key Laboratory of Crop Stress Biology (in Preparation), Hebei Normal University of Science and Technology, Qinhuangdao 066004, China

**Keywords:** *Commelina communis* L., atrazine, resistance, *psbA* gene, antioxidant enzyme

## Abstract

Asiatic dayflower (*Commelina communis* L.) is a detrimental weed that mainly infests corn and soybean fields in China. Recently, some *C. communis* populations have exhibited resistance to atrazine, intensifying the difficulties in controlling the weed. However, little is known on the mechanism underlying *C. communis* resistance to atrazine. Therefore, two populations collected from Jilin (JL-1) and Jiangsu (JS-10) provinces of China were used to evaluate their growth responses to atrazine. The results showed that the JL-1 population displayed a low level of resistance to atrazine compared with JS-10 population, with the resistant index (RI) value of 2.9. To determine if a mutation in the *psbA* gene was the basis for varied resistance to this herbicide, the full-length gene encoding 353 amino acids with no intron was sequenced by using genome-walking techniques. No mutation known to confer resistance to atrazine was observed in either JL-1 or JS-10 populations. The malondialdehyde (MDA) contents relative to the control group were significantly higher in JS-10 population than in JL-1 population at 7 days after treatment with atrazine, suggesting that atrazine induced severer oxidant damage on JS-10 population. Additionally, significantly enhanced activities of antioxidant enzymes, including superoxide dismutase (SOD), catalase (CAT), peroxidase (POD) and ascorbate peroxidase (APX), were detected in the JL-1 population, which was most likely to confer resistance to atrazine. To the best of our knowledge, this is the first investigation into the potential genetic and enzymatic differences contributing to atrazine resistance in this population.

## 1. Introduction

The Asiatic dayflower (*Commelina communis* L.) is one of the most troublesome weeds in China [1]. It widely spreads in the fields of China, with its distribution area increasingly expanding. As a member of the Commelinaceae family, *C. communis* can severely infest corn and soybean fields, causing considerable yield loss [2]. Due to the change in farming systems and lack of effective selective herbicides, this weed species is now the dominant weed in the corn and soybean fields of Northeastern China.

Presently, the management of *C. communis* mainly relies on chemical herbicides because of the high cost of hand weeding. Atrazine (2-chloro-4-ethylamino-6-isopropylamine-1,3,5-triazine), a triazine herbicide, was developed by Basel Geigy Chemical Company (Basel, Switzerland) in 1952 [3,4]. It is a photosystem II inhibitor and competes with plastoquinone for binding to the QB site of D1 protein on the chloroplast thylakoid membrane, thus blocking electron transfer of photosystem II, subsequently causing the production of triplet chlorophyll and single oxygen (^1^O_2_) and finally leading to the death of weeds [5,6,7]. In agricultural practices, this herbicide is widely used in diverse crop fields to control annual grass and broadleaf weeds, particularly *C. communis* [8]. However, a recent study reported that some *C. communis* populations from different geographical locations of China exhibited varied responses to atrazine [2].

In 1970, resistance to atrazine was first reported in *Senecio vulgaris* L. [9]. Since then, a total of 66 weed species have evolved resistance to atrazine, ranking the first place [10]. The mechanisms of resistance to atrazine can be mainly divided into two types: target-site resistance (TSR) and non-target-site resistance (NTSR). *PsbA* gene encodes the target D1 protein of atrazine. Previous works have established that a serine (Ser) 264-glucine (Gly) substitution in D1 protein was responsible for resistance to atrazine in several weed species, such as *Poa annua* L. [11], *Amaranthus hybridus* L. [12] and *Kochia scoparia* (L.) Schrad. [13]. This gene mutation would lead to a lower affinity between the binding sites of D1 protein and atrazine, ultimately decreasing the sensitivity to atrazine [14]. Other mutations in D1 protein, such as Ser264-threonine (Thr), valine (Val) 219-isoleucine (Ile), asparaginate (Asn) 266-Thr, alanine (Ala) 251-Val, phenylalanine (Phe) 255-Ile, leucine (Leu) 218-Val and Phe274-Val substitutions, could also confer resistance to triazine herbicides in weed species [14,15]. As for NTSR mechanisms, enhanced metabolism can endow weed resistance to atrazine. Gronwald et al. [16] found that significantly increased atrazine detoxification via glutathione conjugation was detected in the resistant biotype of *Abutilon theophrasti* Medic compared with the susceptible biotype. It has been noted that resistance of *Amaranthus tuberculatus* (Moq.) J.D. Sauer to atrazine was related with elevated rate of atrazine metabolism [17].

To avoid oxidative damage, plants have evolved various protective mechanisms to reduce and eliminate the reactive oxygen species (ROS) caused by a wide range of stresses [18,19]. Excessive accumulation of ROS, including singlet oxygen (^1^O_2_), superoxide radicals (O_2_·-), hydroxyl radicals (OH·) and hydrogen peroxide (H_2_O_2_), has been observed in plants [20]. Oxidative stress resulting from overproduction of ROS damages the membrane system and some functional biomolecules in plants, and then disturbs physiological and biochemical metabolism balances, leading to inhibited growth, abnormal development and the ultimate death of plants [18]. During this process, the eventual product of membrane lipid peroxidation malondialdehyde (MDA) can be used to indicate the level of lipid peroxidation [20]. The enzymatic antioxidation system, including superoxide dismutase (SOD, EC 1.15.1.1), catalase (CAT, EC 1.11.1.6), peroxidase (POD, EC 1.11.1.7) and ascorbate peroxidase (APX, EC 1.11.1.11), is one of the protective mechanisms to defend against oxidative stress [19]. SOD is known as the first line of defense against ROS damage in organisms, especially scavenging O_2_- [21,22]. POD plays an important role in utilizing a wide range of electron donors to convert H_2_O_2_ to H_2_O, and increased POD activity can prevent plant tissues from injuries caused by herbicides, chemicals and other types of stress [23,24,25]. CAT is found in mitochondria, glyoxysomes and peroxisomes. It mainly participates in removing most respiratory and photorespiratory H_2_O_2_ [20]. In addition, APX, as a rate-limiting enzyme in the ascorbate-glutathione cycle, has a significant role in scavenging H_2_O_2_ [26].

Herbicide stress imparts toxicological effects on weeds by triggering excessive ROS production [27]. Multiple studies have revealed that the ROS scavenging system is associated with weed resistances. For example, Zhao et al. [28] found that ROS elimination in susceptible *Alopecurus aequalis* Sobol. plants was severely damaged or broken by mesosulfuron stress compared with that in resistant plants. A previously reported study showed that an enhanced antioxidant enzyme system enabled atrazine-resistant *Pennisetum americanum* (L.) K. Schum to defend themselves against oxidation damage induced by atrazine [29]. In addition, it has been suggested that the oxygen radical detoxifying system is correlated with *Conyza bonariensis* (L.) Cronq. resistance to paraquat [30]. Atrazine, as a photosystem II inhibitor, would lead to the overproduction of ^1^O_2_ through blocking electron transfer of photosystem II, then resulting in severe oxidant damage on weeds. Based on that, weed resistance to atrazine is likely to be implicated with enhanced abilities to eliminate excessive ROS.

Our previous study showed that some *Commelina communis* L. populations have evolved resistance to atrazine in China [2]; however, the mechanism underlying *C. communis* resistance to atrazine is poorly understood. Therefore, the objectives of the present study were to (1) evaluate the response of two *C. communis* populations (JL-1 and JS-10) to atrazine, (2) ascertain whether there were gene mutations in the *psbA* gene for the studied populations, (3) assay the effects of atrazine stress on membrane lipid peroxidation and (4) compare the activities of well-known antioxidant enzymes (SOD, POD, CAT and APX) in two *C. communis* populations. These results will be highly beneficial to understanding the basis for resistance to atrazine in *C. communis* and may provide new ideas for the establishment of effective control strategies against this weed species.

## 2. Results

### 2.1. Responses of Commelina communis L. to Atrazine

**Figure 1:**
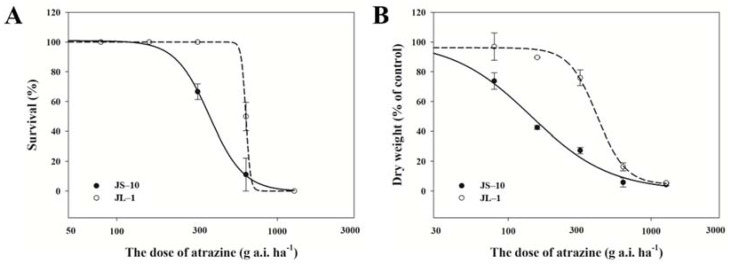
Effects of atrazine on the survival rate (**A**) and growth (**B**) of two *Commelina communis* L. populations (JS-10 and JL-1) at 21 days after treatment.

The JL-1 population exhibited different growth responses to atrazine from JS-10 population (Figure 1). After treatments with 637.5 g a.i. ha^−1^ (half of the recommended dose) of atrazine, the survival rates of JL-1 and JS-10 populations were 49.5% and 11.1%, respectively. The atrazine dose to kill 50% of plants (LD_50_) in the JL-1 population was 637.4 g a.i. ha^−1^, which is 1.7-fold higher than for the JS-10 population (Figure 1A). In addition, the atrazine dose leading to 50% growth inhibition (ED_50_) in the JL-1 population was estimated at approximately 419.9 g a.i. ha^−1^, whereas the JS-10 population had approximately 143.9 g a.i. ha^−1^ (Figure 1B). Based on the resistant index (RI) value, the JL-1 population was 2.9-fold more resistant to atrazine than was JS-10 population and exhibited a low level of resistance to this herbicide.

### 2.2. PsbA Gene Sequencing

**Figure 2:**
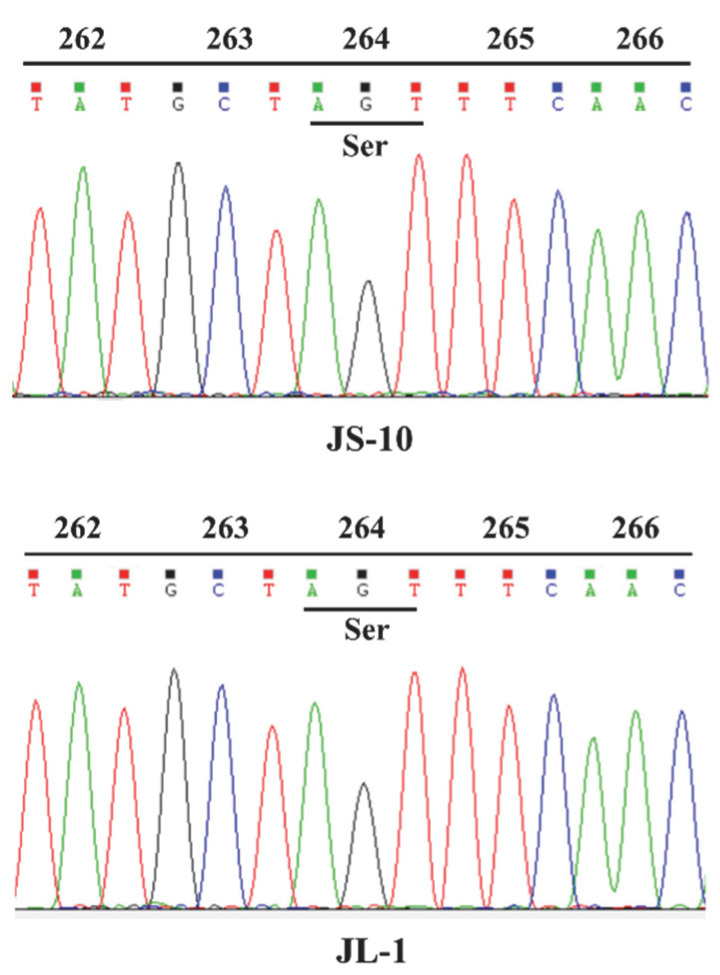
DNA sequencing results showing no mutation in the *psbA* gene in JL-1 population compared with JS-10 population.

A transition from A to G at site 790 (Ser264-Gly substitution) of the *psbA* is the most common point mutation contributing to atrazine resistance in weed species [14]. To determine whether gene mutation in the target protein resulted in the resistance of JL-1 populations to atrazine, full-length *psbA* genes were amplified from 30 individual plants of each population in *C. communis*. Due to no reports regarding the *psbA* gene sequence in *C. communis*, its complete *psbA* gene sequence was obtained with genome walking techniques. The full-length *psbA* gene (GenBank accession number, MN239110) of *C. communis* consisted of 1062 bp and encoded 353 amino acids with no intron detected. None of the samples sequenced showed amino acid substitution at 264 or other sites (Figure 2 and Supplementary Materials Figure S1), indicating that resistance to atrazine in the JL-1 population did not result from the gene mutation in the target protein.

### 2.3. Effect of Atrazine Stress on Lipid Peroxidation

**Figure 3:**
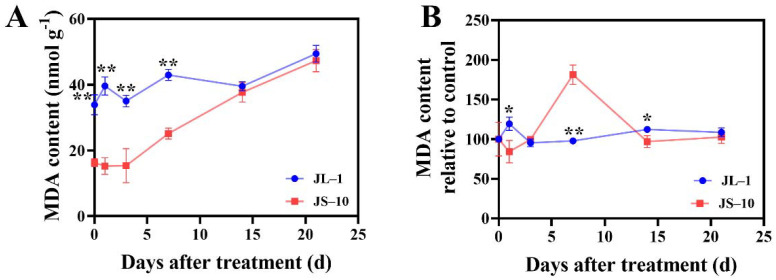
Malondialdehyde (MDA) contents (**A**) and the percent of MDA contents compared to control group (**B**) in two *Commelina communis* L. populations (JS-10 and JL-1) at 0, 1, 3, 7, 14 and 21 days after atrazine spray. * *p* < 0.05; ** *p* < 0.01.

The MDA levels in the JS-10 population were relatively stable at the beginning and then sharply increased with time, from 3 days after treatment (DAT) to 21 DAT; however, for the JL-1 population, its contents remained at a relatively stable level after spraying with atrazine. Compared with the JS-10 population, the JL-1 population exhibited higher MDA contents (*p* < 0.01) throughout the whole experimental period except at 14 DAT and 21 DAT (Figure 3A). The MDA content relative to the control in the JL-1 population slightly increased from 0 to 1 DAT, followed by declining back to the initial level and then remaining stable. The JS-10 population had a maximum level at 7 DAT and subsequently decreased to a lower level than that present in the JL-1 population. At 7 DAT, the MDA content relative to the control in the JS-10 population was significantly higher than that in the JL-1 population (*p* < 0.01); nevertheless, the opposite tendency was observed at 1 and 14 DAT (*p* < 0.05) (Figure 3B).

### 2.4. Antioxidant Enzyme Activity under Atrazine Stress

**Figure 4:**
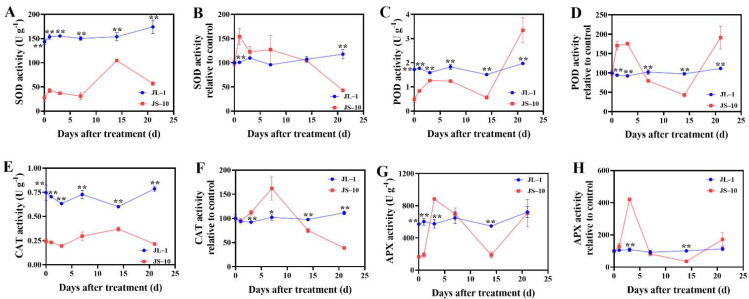
Activities of antioxidant enzymes (**A**,**C**,**E**,**G**) and the percent of antioxidant enzyme activities compared to control group (**B**,**D**,**F**,**H**) in two *Commelina communis* L. populations (JS-10 and JL-1) at 0, 1, 3, 7, 14 and 21 days after atrazine spray. * *p* < 0.05; ** *p* < 0.01. SOD, superoxide dismutase; POD, peroxidase; CAT, catalase; APX, ascorbate peroxidase. Other figures are as shown in Figure 3.

The SOD activity of the JS-10 population reached two peaks at 1 and 14 DAT. For the JL-1 population, the change dynamics in SOD activity were not obvious in response to atrazine. During the entire experimental period, distinctly enhanced SOD activity was detected in the JL-1 population in comparison with the JS-10 population (*p* < 0.01) (Figure 4A). Relative to the control group, the SOD activity of the JL-1 population was relatively stable, whereas, for the JS-10 population, it increased to a noticeably higher level (*p* < 0.01) and then gradually decreased to a lower level (*p* < 0.01) than observed in the JL-1 population (Figure 4B). The POD activity and POD activity relative to the control for the JS-10 population initially increased, then decreased and finally rose to the highest level; however, the JL-1 population showed stable levels when exposed to atrazine. A clear increase in POD activity was observed in the JL-1 population compared when with the JS-10 population, except at 21 DAT (*p* < 0.01) (Figure 4C,D). Similar to SOD, the CAT activities of the JS-10 population were always lower (*p* < 0.01) than those of the JL-1 population from 0 to 21 DAT (Figure 4E). The percentage of CAT activity compared to the control group in the JL-1 population remained steady throughout the whole experimental period. In contrast, that of the JS-10 population visibly increased from 0 to 7 DAT and then gradually decreased to a lower level (*p* < 0.01) than that detected in the JL-1 population (Figure 4F). Both APX activity and APX activity relative to the control in the JL-1 population exhibited a relatively stable level after treatment with atrazine. For the JS-10 population, the APX activity increased from 0 to 3 DAT, declined from 3 to 14 DAT and then rose to the same level as that observed in the JL-1 population. A similar trend was found in the percentage of APX activity relative to the control group. In addition, APX activity and its percentage relative to the control group in the JL-1 population were 2.91- and 2.84-fold higher than those in the JS-10 population, respectively (Figure 4G,H). Collectively, the responses of antioxidant enzymes to atrazine were more obvious in the JS-10 population than in the JL-1 population. Additionally, the JL-1 population showed enhanced antioxidant enzyme activity compared with the JS-10 population when exposed to atrazine.

## 3. Discussion

*C. communis*, with a well-developed root system, has strong regeneration ability and extensive adaptability to the environment, and these attributes contribute to its tolerance to herbicide stress. A previous study showed that this weed species is less sensitive to several herbicides, such as acetochlor, fluroxypyr and glyphosate, popularly used in corn and soybean fields, [31]. Atrazine is widely used for the control of *C. communis*, particularly in Northeastern China. In present study, the JL-1 population evolved a low level of resistance to atrazine compared with the susceptible population JS-10. The JL-1 population was collected from corn fields with continuous use of atrazine for over 35 years in Jilin Province, located in Northeastern China. Additionally, it is surveyed that atrazine was used in Northeastern China at the dose of approximately 3000 g a.i. ha^−1^, which is 2.4-fold higher than the recommended dose [32]. In contrast, the JS-10 population was collected from corn fields with no records of atrazine use in Jiangsu Province. Thus, it is speculated that a long history and excessive level of atrazine use contributed to the evolution of *C. communis* resistance to this herbicide.

Gene mutation in the *psbA* gene was the main TSR mechanisms for weed resistance to photosystem II inhibitors. The most common amino acid substitution of the *psbA* gene (Ser264 -Gly) has since been documented in many weed species. Hirschberg and McIntosh [12] reported that Ser264-Gly substitution in D1 protein conferred a high level of resistance to s-trazine in *Amaranthus hybridus* L. Svyantek et al. [11] found that Ser264-Gly mutation in the *psbA* gene was one of the reasons for resistance to atrazine, amicarbazone and diuron in *Poa annua* L. Other than Ser264-Gly substitution, it has been reported that Val to Ile mutation at 219 codon of D1 protein resulted in *P. annua* resistance to metribuzin and diuron [15]. Beyond that, resistance to photosystem II inhibitors due to amino acid mutations in D1 protein has also been observed in *Kochia scoparia* (L.) Schrad., *Chenopodium album* L., *Sisymbrium orientale* L. and *Amaranthus powellii* S. Watson [13,33,34,35]. However, compared with the JS-10 population, no gene mutation was detected in the whole *psbA* sequence of JL-1 population. Therefore, there may be other explanations for the resistance to atrazine in the JL-1 population.

Cell structures, such as lipids, membranes, proteins and nucleic acids, are vulnerable to damage by ROS. Excessive ROS might lead to lipid peroxidation, enzyme passivation, protein denaturation and even genetic mutation [36,37]. The MDA content in the JS-10 population markedly increased after spraying with atrazine, as is consistent with previous findings that atrazine could induce MDA accumulation in *Zea mays* L. [38]. However, a slight increase in MDA content was detected in the JL-1 population. Compared with the control group, a drastic accumulation of MDA was observed in the susceptible JS-10 population at 7 DAT, indicating that atrazine imposed a severe oxidant damage on the JS-10 population. By contrast, no distinct difference was detected in the resistant JL-1 population, suggesting that it has a strong ability to resist oxidant stress. Similarly, no significant difference in MDA content was determined between atrazine treatment and control groups in tolerant *P. americamum* as well [29]. Given the above, in comparison with the resistant JL-1 population, more atrazine-induced ROS might accumulate in the susceptible JS-10 population, leading to serious lipid peroxidation and ultimately inhibiting plant growth.

Generally, plants protect themselves against the damage caused by abiotic stresses, such as ROS-induced oxidative damage, via multiple defense systems, including antioxidant enzyme defense systems and non-enzymatic antioxidants [23,39]. SOD is an important antioxidant enzyme responsible for catalyzing the disproportionation of two O_2^−^_ radicals to H_2_O and O_2_ to relieve the injury induced by ROS [40]. POD, CAT and APX are critical for converting H_2_O_2_ to H_2_O and O_2_ to reduce ROS poisoning. In the present study, the activities of antioxidant enzymes, particularly SOD and CAT, were clearly higher in the JL-1 population than in the JS-10 population when exposed to atrazine. Therefore, ROS induced by atrazine stress might be eliminated quickly in the JL-1 population, which would alleviate the damage to biomacromolecules and photosynthetic organs caused by excessive ROS. Given that, the high antioxidant ability of the JL-1 population is most likely to be responsible for its resistance to atrazine. It has been reported that resistant *A. aequalis* plants exhibited enhanced ROS scavenging in comparison with sensitive biotypes [28], thus largely supporting the above speculation. Additionally, an increased antioxidant system could confer higher tolerance to atrazine in *Z. mays* and *Oryza sativa* L. [38,41]. Further analysis found that atrazine had a slight effect on the antioxidant enzymes of the JL-1 population. However, the activities of the studied antioxidant enzymes increased at the beginning in the JS-10 population after treatment with atrazine, meaning that the JS-10 population defends itself from atrazine stress by strengthening the antioxidant enzyme system. Subsequently, its antioxidant enzyme activities gradually declined with time, suggesting that this population finally cannot withstand the oxidant stress caused by atrazine. Moreover, at 0 DAT, the JS-10 population displayed weaker activities of antioxidant enzymes than the JL-1 population, indicating that the strong ability to resist oxidant damage in the JL-1 population may not be induced by atrazine spray. Atrazine was used in corn fields where the JL-1 population was collected for approximately 35 years, whereas this herbicide was never used in locations where the JS-10 population was collected; this might be one of the reasons for the JL-1 population having evolved high antioxidant abilities.

## 4. Materials and Methods

### 4.1. Chemical Reagents

Atrazine (50% suspended concentration) was provided by Shandong Binnong Technology Co., Ltd. (Shandong, China).

### 4.2. Plant Materials and Growth Condition

*C. communis* seeds were collected from corn fields located in the Jilin (JL-1) and Jiangsu provinces of China (JS-10). Mature seeds were randomly collected from at least 200 plants via an inverted “W” pattern. To break seed dormancy, the endosperm cap was carefully removed from each seed before sowing *C. communis*. The seeds were germinated in perish dishes containing two layers moistened with sterilized water after washing three times with sterilized water. Then, all the dishes were put into a growth chamber under 60% relative humidity (RH) and 25 °C/20 °C (day/night), with a 12-h photoperiod of 300 μmol m^−2^s^−1^. The germinated seeds were transplanted into plastic pots (10.5 cm × 9 cm) containing a mixture of soil and organic fertilizer (3:1, *v*:*v*). Subsequently, the pots were put in a glasshouse (natural light, 30 °C/25 °C, 60% RH) and watered every two days. Seedlings were thinned to five uniform plants per pot when they reached the one-leaf stage.

### 4.3. The Responses of Commelina communis L. to Atrazine

When the *C. communis* seedlings reached the three- to four-leaf stage, they were sprayed with atrazine at concentrations of 79.69, 159.38, 318.75, 637.5 and 1275 g a.i. ha^−1^, using an ASS-3 Walking Spray Tower (450 L ha^−1^; National Engineering Research Center for Information Technology, Beijing, China). The concentration of 1275 g a.i. ha^−1^ is the field-recommended application rate. Plants treated with distilled water were considered as the control. Then, all the plants were placed in a glasshouse under the same conditions as described above. At 21 DAT, the dry weight of the aboveground seedlings was measured and expressed as a percent of control group plants. Each treatment included five pots, each of which contained five seedlings, and the experiment was independently repeated three times.

### 4.4. psbA Gene Clone

**Figure 5:**
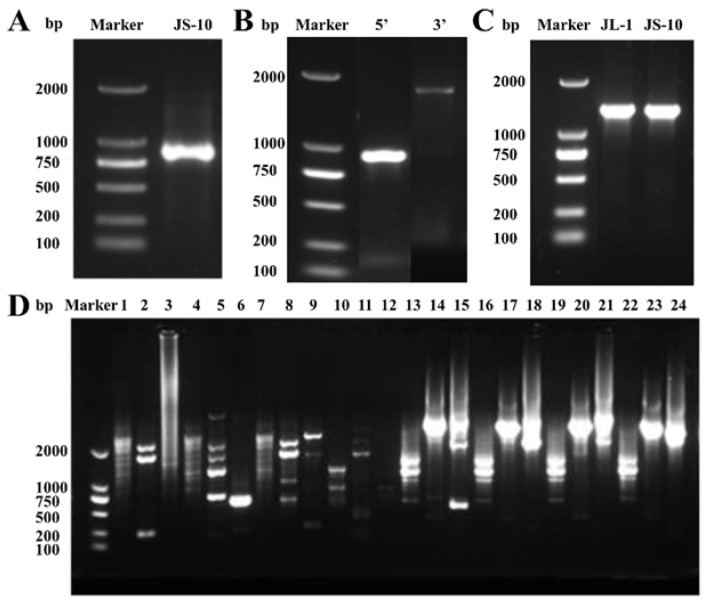
(**A**) Agarose gel electrophoresis of the PCR products of partial *psbA* gene amplification. (**B**) Agarose gel electrophoresis of 5′ and 3′ end clones of *psbA* gene. (**C**) Agarose gel electrophoresis of full-length *psbA* gene in JL-1 and JS-10 populations. (**D**) Agarose gel electrophoresis of the amplifications of 5′ and 3′ ends of *psbA* gene; 1–12 represent the amplicons by AP1, AP2, AP3 and AP4 with 5′-SP1, 5′-SP2 and 5′-SP3 in 5′ end, respectively; 13–24 represent the amplicons by AP1, AP2, AP3 and AP4 with 3′-SP1, 3′-SP2 and 3′-SP3 in 3′ end, respectively.

Fresh leaves were collected from JS-10 populations at the three- to four-leaf stage. Total DNA was isolated with a Tiangen DNAsecure Plant Kit (Tiangen Biotech Co., Ltd., Beijing, China). Primers (1F/1R) to amplify the partial *psbA* gene sequence were designed based on the conserved regions of the *psbA* gene in *O. sativa* (GenBank accession number, NC_031333.1) and *Arabidopsis thaliana* (L.) Heynhold (GenBank accession number, NC_000932.1) with Primer Premier 5 (Primer Biosoft International, Palo Altro, CA) (Table 1). PCR was conducted in a total volume of 25 μL containing 1 μL of DNA, 1 μL of forward and reverse primer (1.0 × 10^−8^ mol L^−1^), 12.5 μL of 2× Taq PCR Master Mix (Tiangen, Beijing, China) and 9.5 μL of double-distilled water (ddH_2_O). Amplification cycling began with a denaturation step for 1 min at 95 °C, followed by 35 cycles of 94 °C for 1 min, 55.4 °C for 1 min, 72 °C for 70 s and a final extension step at 72 °C for 10 min. PCR products were evaluated with 1.0% agarose gels (Figure 5A) and then sequenced by Beijing Genomics Institution Technology (Beijing, China).

Six primers (3′SP1, 3′SP2, 3′SP3, 5′SP1, 5′SP2 and 5′SP3) were designed based on the obtained partial sequence of the *psbA* gene and the primer design instructions of the Genome Walking Kit (TaKaRa Biotechnology, Dalian, China; Table 1). The 5′ and 3′ end sequences of the *psbA* gene in *C. communis* were amplified from the two ends of the obtained sequence according to the protocol of the Genome Walking Kit. The PCR products were detected with 1% agarose gels after each step of PCR amplification (Figure 5D). According to the length of amplification fragment, degeneracy primers AP2 and AP3 were suitable for amplifying 5′ and 3′ends of *psbA* gene, respectively. Then, products of the third PCR amplification were cloned into a T1 vector based on the manufacturer’s protocol of the *pEASY^®^*-T1 Cloning Kit (TransGen, Beijing, China), followed by transformation into competent *Escherichia coli* cells. The positive clones were selected (Figure 5B) and sequenced by Beijing Genomics Institution Technology. The sequencing results were analyzed and assembled, using DNAMAN software (Lynnon LLC, San Ramon, CA, USA).

### 4.5. psbA Gene Sequencing

To determine whether there was a mutation in the *psbA* gene, the target fragment was amplified in the JS-10 and JL-1 populations. Thirty plants of each population were selected and used for DNA extraction. For JL-1 populations, surviving plants at 637.5 g a.i. ha^−1^ were selected. Based on the obtained complete *psbA* gene sequence, a pair of specific primers (2F/2R) was designed to amplify the full length of the *psbA* gene (Table 1). PCR amplification was performed as described above, with an annealing temperature and a final extension time of 51.5 °C and 100 s, respectively. The PCR products were evaluated visually with a 1% agarose gel (Figure 5C) and then sequenced. The sequencing results were analyzed by using DNAMAN software.

### 4.6. Malondialdehyde Content Assay

Young leaves of distilled water- or atrazine-treated (159.38 g a.i. ha^−1^) plants were collected from JL-1 and JS-10 populations at 0, 1, 3, 7, 14 and 21 DAT. Five hundred milligrams of leaf tissue was frozen and then ground into a fine powder with liquid nitrogen. Sample powders were extracted in 4.5 mL of precooled 10% (*w*/*v*) trichloroacetic acid (TCA) before centrifugation at 12,000× *g* and 4 °C for 10 min. The supernatant (2 mL) was mixed with an equal volume of 0.6% (*w*/*v*) thiobarbituric acid (TBA). Then, the mixture was heated in boiling water for 15 min, followed by rapid cooling in an ice-water bath. The OD values of the supernatant were evaluated at 532, 600 and 450 nm after centrifugation at 12,000× *g* and 4 °C for 15 min. The MDA content was calculated based on the method reported by Liu et al. [42].

### 4.7. The Assay of Antioxidant Enzyme Activities

Samples for the MDA content assay were also used for the determination of antioxidant enzyme activity. Fresh leaves were frozen in liquid nitrogen and then ground into a fine powder. Powered samples (200 mg) were extracted in 1.8 mL of precooled phosphate buffer (PBS, 0.05 mol L^−1^, pH = 7.8). The supernatant was obtained after centrifugation at 4 °C and 12,000× *g* for 10 min and then used for antioxidant enzyme activity assays. The activities of SOD, POD, CAT and APX were determined according to the manufacturers’ instructions for SOD, POD, CAT and APX Enzyme-Linked Immunosorbent Assay Kits (Kenuodi Biotechnology Co., Ltd., Quanzhou, China), respectively.

### 4.8. Statistical Analysis

All experimental data are expressed as the mean ± standard deviation (SD). In the whole-plant dose-response experiment, the data were subjected to a log-logistic non-linear regression model, using SigmaPlot version 12.5 software (Systat Software, Point Richmond, CA, USA) [43]:

*Y* = *C* + (*D* − *C*)/[1 + (*X*/*X_0_*)*^b^*] (1)

where *C* and *D* are the lower and upper limits, respectively; *X* represents the herbicide dose; *X_0_* is the herbicide dose required for 50% growth inhibition (ED_50_); *b* is the slope of the curve; and *Y* represents the growth response at herbicide dose *X*. The resistant index (RI) was calculated as follows:

RI = ED_50_ (resistant population)/ED_50_ (susceptible population) (2)

In the MDA content and antioxidant enzyme activity assay experiment, a *t*-test was employed to analyze the significant differences between the JS-10 and JL-1 populations (*p* < 0.05) via SPSS version 21.0 software (IBM Crop, New York, NY, USA).

## 5. Conclusions

Collectively, the JL-1 population has evolved a low level of resistance to atrazine in comparison with the JS-10 population. Gene mutation in the *psbA* gene was not the reason for resistance to atrazine in the JL-1 population. Severe lipid peroxidation caused by atrazine was detected in the JS-10 population, in contrast with JL-1 population. Compared with the JS-10 population, a clear increase in antioxidant enzyme activities was observed in the JL-1 population, conferring a low level of resistance to atrazine. These findings will help to better understand the mechanisms underlying resistance to atrazine in *C. communis* and provide new insights into establishing diverse and effective control strategies against this weed species aimed at different geographic areas.

## Figures and Tables

**Table 1:** Information on the primers used in the study.

Primer Name	Sequence (5′-3′)
1F	ATCGGATGGTTCGGTGTT
1R	GAGGGAAGTTGTGAGCATTACG
2F	CTATTGAGATTGGTTGACATT
2R	TGGTTTATTCCGCAGCAGCA
5′SP1	GCTAAGTTCCCACTCACGACCCAT
3′SP1	ACAGAAAACGAGTCCGCAAATGA
5′SP2	ACCGCCGTTGTATAACCACTCATC
3′SP2	GTTTCAACAACTCTCGTTCTTTACAC
5′SP3	AGGAATAATGGCACCAGAGATAA
3′SP3	CCAATCTGTAGTTGATAGTCAGGGGC

## Data Availability

The sequence of *C. communis psbA* gene was deposited in the NCBI with GenBank accession number of MN239110.

## Supplementary Materials

The following are available online at https://www.mdpi.com/article/10.3390/plants10122685/s1, Figure S1: Sequence alignment of psbA gene between JL-1 and JS-10 in *Commelina communis* L. Red box and blue box represent initiator codon and terminator codon, respectively.
