# Supplementary material for: High Antioxidant Ability Confer Resistance to Atrazine in Commelina communis L
Source: Plants (Basel). 2021 Dec 7;10(12):2685. doi: 10.3390/plants10122685 (PMC8707497; doi:10.3390/plants10122685)
Supplement: Supplementary file 1 [file plants-10-02685-s001.zip › plants-1493050-supplementary.pdf]

Supplementary materials

Title: High antioxidant ability confer resistance to atrazine in *Commelina communis* L.

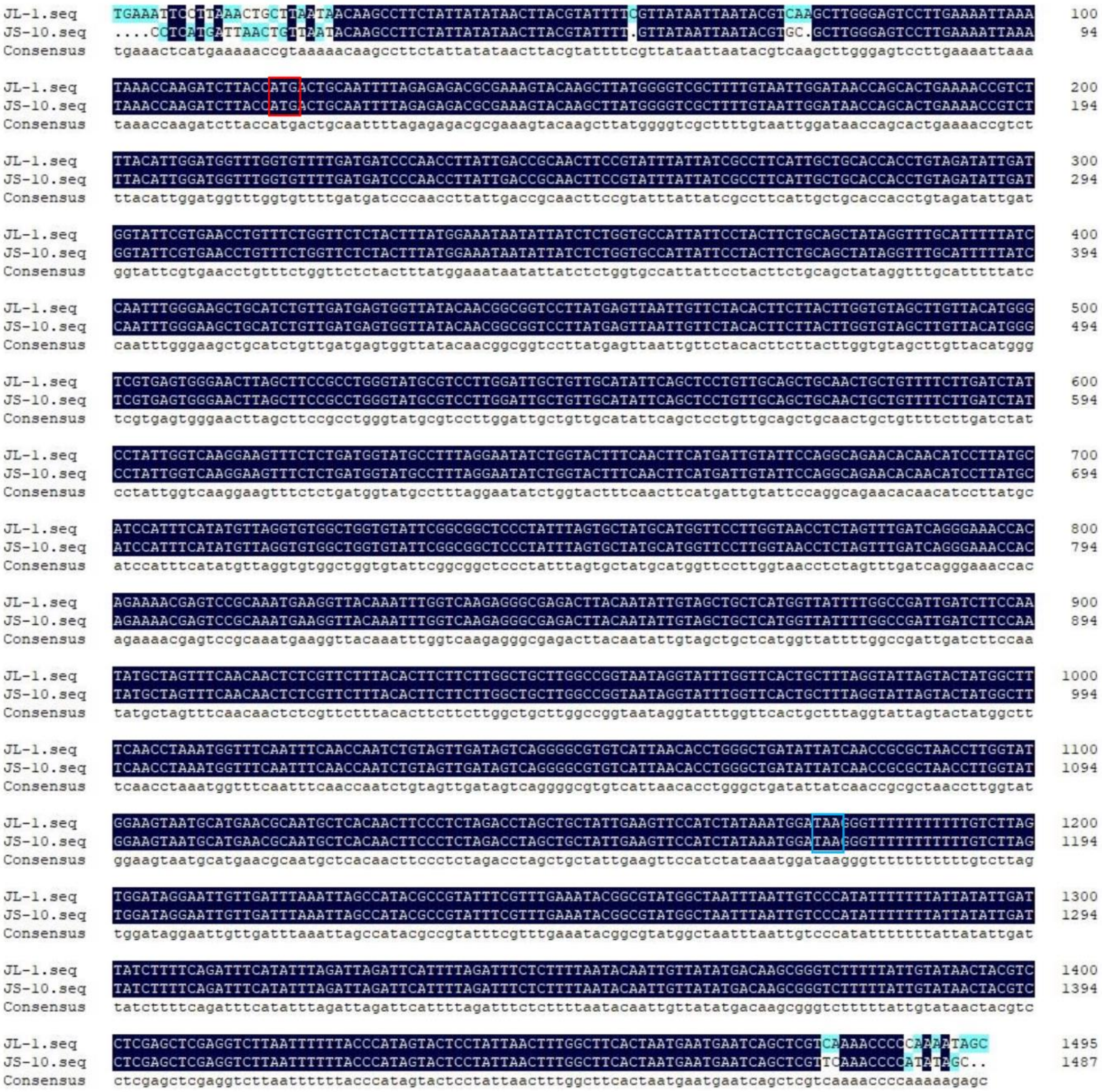

Figure S1 Sequence alignment of *psbA* gene between JL-1 and JS-10 in *Commelina communis* L.. Red box and blue box represent initiator codon and terminator codon, respectively.
